# Clients of VA-Housed Legal Clinics: Legal and Psychosocial Needs When Seeking Services and Two Months Later

**DOI:** 10.21061/jvs.v6i1.167

**Published:** 2020-07-29

**Authors:** Christine Timko, Jack Tsai, Emmeline Taylor, David Smelson, Daniel Blonigen, Amia Nash, Andrea Finlay

**Affiliations:** 1 Department of Veterans Affairs, US; 2 University of Colorado-Colorado Springs, US

**Keywords:** Veterans, civil legal services, VA-housed legal clinics

## Abstract

Veterans often need civil legal services, yet little is known about veterans’ use and consequences of these services. This study examined veterans seeking legal services at VA-housed legal clinics. Baseline data from 61 clients of two VA-housed legal clinics were used to identify clients’ legal needs and psychosocial characteristics. Data collected from 49 (80%) of the same clients two months later were used to address clients’ improvement and satisfaction after receiving legal services. At baseline, clients reported a mean of 6.0 (SD = 4.2) legal needs, with the most common being help obtaining VA benefits (87%). Clients represented a vulnerable population in that most had an extensive criminal history (e.g., had been arrested, charged, and incarcerated) and multiple health care needs (had a chronic medical condition, had recently received treatment in an emergency department, and had received psychological treatment due to significant psychological symptoms). At follow-up, clients reported a mean of 4.4 (SD = 3.8) legal needs. Tests to identify changes between baseline and follow-up on legal needs, housing arrangement, psychological symptoms, and substance use yielded few significant results. Most participants did not receive additional help with their legal matters after the baseline appointment. At follow-up, clients reported that few of their legal needs were met but also that they were mostly satisfied with the legal services they received. Findings suggest that because clients may need more intensive legal intervention of longer duration to resolve their legal needs and achieve better housing and health status, VA-housed legal clinics require greater resources and expansion.

Legal clinics housed at the Department of Veterans Affairs (VA) facilities were developed to help veterans address civil legal issues that are barriers to receiving health and social services. In the US, individuals who face civil legal issues do not have a constitutional right to legal counsel, and so must secure paid counsel or obtain free or pro bono civil legal assistance. Because the civil legal system was designed to require an attorney in most legal situations, civil legal aid programs such as VA-housed legal clinics have been the predominant source of legal assistance to underserved and vulnerable populations such as veterans (Keith, 2016). In 2011, 1.7 million Americans who were eligible for civil legal assistance were military veterans (Keith, 2016).

Statewide studies of veterans suggest that their legal needs often stem from problems related to homelessness or housing instability, or access to benefits and needed health services to manage PTSD or military sexual trauma (Farmer et al., 2017; State Bar of California, 2019). The first VA-housed legal clinic was opened in 2008 at the Houston VA in response to growing awareness that receipt of legal services may prevent veterans’ homelessness. Although VA cannot provide legal services, VA medical centers can provide space to host non-VA legal clinics. VA-housed legal clinics are mainly independent from VA, with services provided by law schools or non-profit organizations (Timko et al., 2019). A nationwide survey of VA-housed legal clinics found that 61% of clinics were unable to serve most veterans seeking legal services due to lack of clinic funding and staff time (Timko et al., 2019). Many legal clinics had been operating for relatively few years (M = 4.0 years, SD = 2.6), which is indicative of their recent major expansion. Most VA-housed clinics were located in urban or suburban areas (79%), suggesting that rural veterans in need of legal services may not be within driving distance of a VA-housed clinic. VA-housed legal clinics are generally not in fully integrated medical-legal partnerships (MLPs), which have formalized agreements and coordination between medical and legal staff, and have been found to improve patients’ health outcomes and save money for both patients and health care systems (Tuller, 2016). Veterans also receive legal services in settings other than VA-housed legal clinics such as through the National Law School Veterans Clinic Consortium (NLSVCC), which is a collaborative effort of US law school legal clinics to address the unique legal needs of veterans on a pro bono basis.

This study examined veterans seeking legal services at two VA-housed legal clinics. It asked three questions: (1) What are the legal needs and psychosocial characteristics of veterans using VA-housed legal clinics? (2) Do veterans’ legal and psychosocial needs improve after using legal clinic services? (3) Are clients satisfied with the legal services they received? The purpose was to inform VA-housed legal clinics on how they may improve services to better meet clients’ needs and preferences.

## Legal Needs and Psychosocial Characteristics of Clients Using Legal Clinics

A report by the White House Legal Aid Interagency Roundtable’s Civil Legal Aid Research Workshop concluded that research activity on civil legal services has been limited and fragmented. It urged the use of existing data from smaller projects that are already being conducted to inform better legal service provision (Jweied & Yang-Green, 2016). In keeping with the report’s conclusion, we found little research that has examined the characteristics of legal clinic clients.

In one study, Tsai et al. (2017a) collected data on 791 veterans served by four MLPs. Veterans presented to MLPs with over ten types of legal matters: consumer, criminal, employment, estate or probate, family, housing, military, public benefits, tax, VA benefits, and other concerns. Data gathered at two of the four study sites showed that the top five legal matters were VA benefits (27%), housing (e.g., evictions; 19%), family (e.g., divorce or child support; 13%), consumer (e.g., debt; 12%), and public benefits (e.g., Social Security; 8%). Similarly, among recipients of free civil legal services in the State of California, housing, family, consumer, and income maintenance were among the most common types of needs addressed (State Bar of California, 2019).

On veterans’ characteristics, Tsai et al. (2017a) found that the mean age of MLP clients was 52 (SD = 13), most were male (87%), 47% were white and 36% were black, and 22% were married while 36% were divorced or separated. In addition, most (57%) reported a lifetime history of homelessness, 30% served in a combat zone, and 20% served in Iraq or Afghanistan. Further, 40% had a diagnosis of PTSD, 16% had a substance use disorder, 15% had a schizophrenia-spectrum disorder or bipolar disorder, and 8% had major depression. A study by Renner and Hartley (2018) of women civilians using civil legal services in response to victimization by intimate partner violence similarly found that at baseline many clients reported psychological symptoms that raised clinical concerns; 67% met criteria for depression, and 64% met criteria for PTSD. Together, these studies suggest that veterans using civil legal services may have not only an array of legal needs but social services and mental health needs as well.

## Do Legal and Psychosocial Needs Improve After Use of Legal Clinic Services?

As pointed out by Shanahan, Selbin, Mark, and Carpenter (2018) regarding law school clinics, although many service providers believe that legal clinics’ clients are better off with assistance than without, there is limited empirical evidence to support these views. Tsai et al. (2017a), in their one-year study of veterans who accessed legal services at MLPs, observed significant improvements in housing, income, and mental health. Veterans who received more MLP services showed greater improvements in housing and mental health than those who received fewer services, and those who achieved their predefined legal goals showed greater improvements in housing status and community integration than those who did not. Similarly, Renner and Hartley’s (2018) one-year study of women who experienced intimate partner violence victimization and received civil legal services found that participants reported a decrease in mental health symptoms (depression and PTSD).

## Are Clients Satisfied with Legal Clinic Services?

We were unable to find any published studies of legal clinic clients’ satisfaction with services they received, despite the Legal Services Corporation’s (LSC) call for such studies. The LSC was created by Congress in 1974 to provide financial support for civil legal aid to low-income Americans. As explained by the LSC (2007), legal clinic performance criteria should include client satisfaction as an important outcome in clinic evaluations. Assessing this outcome will help the clinic to determine whether it is responsive enough and has procedures in place to recognize and adjust to new, emerging needs of its target population and to allocate existing resources accordingly.

In summary, this study initiated steps to address knowledge gaps about civil legal services in light of the sparse published work in this field. These gaps include knowledge about the characteristics of clients using these services, the extent to which using these services helps to lessen clients’ legal and psychosocial needs, and how satisfied clients are with the legal services they receive. We used a longitudinal study design to address these aims.

### Current Study

The current study collected data from 61 clients of VA-housed legal clinics at baseline, that is, when they initially sought legal services from the clinic. These data were used to answer our first question of identifying clients’ legal needs and psychosocial characteristics. Two months later, we collected data from 49 (80%) of the same clients. These data were used to address the questions of clients’ improvement and satisfaction after receiving legal services. The study’s aim was to begin to fill in research gaps as to how veterans’ legal clinics may better meet their mission to address legal needs that may reduce access to needed health and social services.

## Methods

### Sample and Procedure

The sample was composed of 61 clients of two VA-housed legal clinics. Both clinics were located in the San Francisco Bay Area, about 35 miles apart. One was located in a suburban area, and one in an urban area; both offered pre-arranged and walk-in appointments one day per month. These sites were selected because of their location; that is, they were within driving distance of the research team to allow travel to regular in-person meetings with staff and clients. To accrue the sample, clients who appeared at the clinics were approached consecutively by study staff who explained the project and asked the potential participant to provide informed consent. Of 77 clients approached about the study, 61 (79%) provided informed consent and the baseline assessment. The baseline assessment was completed by participants in the waiting area of the legal clinic. The study team attempted to contact each participant by telephone for a two-month follow-up; 80% (n = 49) completed the follow-up assessment.

Client demographic characteristics at baseline are shown in Table 1. The sample was mostly male, from racial minority groups, unemployed, unmarried, and had stable housing. The mean age was 57 years old, the mean education level was the equivalent of two years of college, and the mean monthly income reported yielded a calculated income of about $13,000 per year. About one-half of participants had served in the Army, about one-third had served in a combat zone, and most had received an honorable discharge, although over one-half had received some disciplinary action while in the military. The mean number of years having served in the military was five. Baseline demographic characteristics did not differ between participants who completed or did not complete the follow-up assessment.

### Measures

**Figure 1:**
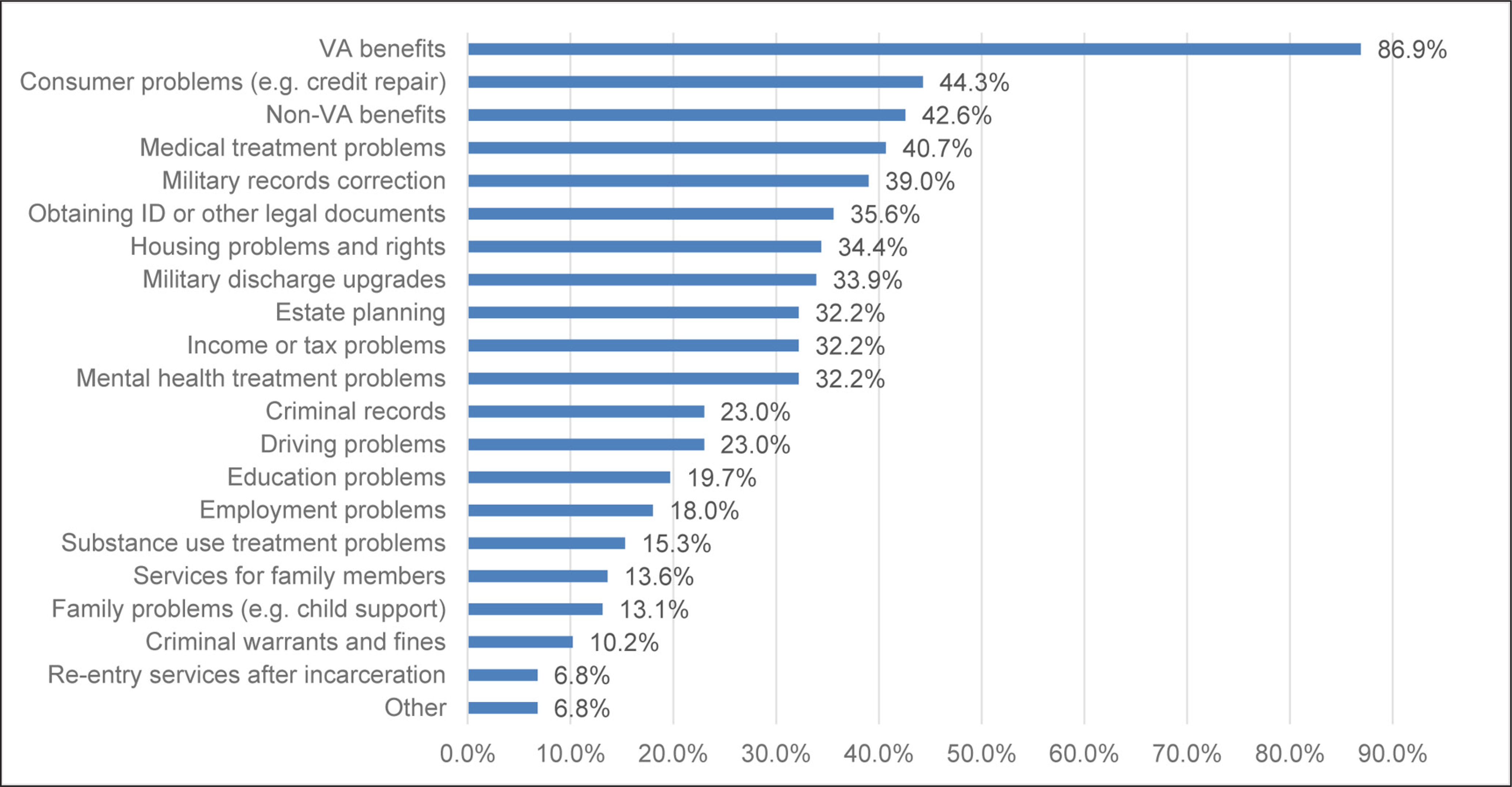
Types of legal help needed at baseline (n = 61).

The baseline and follow-up questionnaires that were developed and pretested used selected portions of the Addiction Severity Index, which is a structured, clinical research assessment that has been used to monitor veterans’ outcomes (Leonhard et al., 2000; Moos et al., 2000). At baseline, the questionnaire completed by participants included demographics (15 items; see Table 1); their legal needs (21 items; see Figure 1); and their psychosocial characteristics, including history of criminal justice involvement, health and mental health status, substance use, and health care utilization (36 items; see Table 2). At the two-month follow-up, the questionnaire completed by participants included the same items as at baseline on legal needs, mental health status, and substance use; and items on how much legal help was obtained and satisfaction with that help (9 items; see Table 3).

### Data Analysis

Data analysis at baseline consisted of obtaining descriptive statistics (n = 61). Specifically, for nominal and categorical variables, frequencies and percentages are reported. For continuous variables, means and standard deviations are reported. To analyze change over time, we conducted McNemar tests (two-sided) to examine participants’ reports of change between baseline and the two-month follow-up (n = 49). SPSS, which we used to conduct analyses, does not give the value of the McNemar chi-square, only its p-value.

## Results

### Clients' Civil Legal Needs at Baseline

Figure 1 shows results for clients’ reports of their legal needs at the time they sought services. The most commonly needed legal service was help applying for and obtaining VA benefits (87%). Over 40% of clients wanted help with consumer problems (such as debt and credit repair), applying for and obtaining non-VA benefits (such as Social Security or Social Security Disability Income), and problems with obtaining medical treatment (such as care access or payment). Roughly one-third of clients wanted help with correcting their military records, obtaining ID and/or other legal documents, housing problems and rights (such as preventing eviction and foreclosure), military discharge upgrades (which change the “character of service” shown on the military discharge certificate, e.g., from general to honorable discharge; see note on Table 1), estate planning (such as wills, advance health care directives, and durable powers of attorney), income or tax problems, or problems obtaining mental health treatment. About one-quarter of clients (23%) wanted help with their criminal records (such as expungement, sealing, or correction) or driving problems (such as driver’s license restoration). Twenty percent or less wanted help with education problems, employment problems (such as worker’s compensation), substance use treatment problems, legal services for family members, or family problems (such as child support, custody, or divorce). Finally, 10% or less wanted help with criminal matters (such as outstanding warrants and fines), re-entry services after incarceration, or other legal difficulties. At baseline, the mean number of kinds of legal needs reported by participants was 6.0 (SD = 4.2; range = 1–16).

### Clients' Psychosocial Characteristics at Baseline

#### Criminal Justice Involvement

At the time of seeking VA-housed legal services, 4.9% of clients were awaiting charges, trial, or sentencing, 8.3% were on parole or probation, and 3.3% were participating in a treatment court (Table 2). The majority had a lifetime history of being arrested and charged (82.0%) and incarcerated (60.7%). Among those with a history of incarceration, the mean number of months incarcerated was 25.8 (SD = 42.2).

#### Health

The majority of participants had a chronic medical condition (78.7%) for which they were taking prescribed medication (65.6%). In addition, the majority had a lifetime history of having received psychological treatment (80.3%), and 5.0% received a psychiatric disability pension. Consistently, as shown in Table 2, the majority of participants had a lifetime history of having had a significant period of time experiencing serious depression, serious anxiety, trouble understanding, and serious thoughts of suicide. About three-quarters of participants (73.8%) had taken prescribed medication for a psychological condition in their lifetime. These findings for lifetime mental health problems were reflected in clients’ problems in the 30 days before seeking legal services, during which the majority had serious depression, serious anxiety, and trouble understanding, and were taking prescribed medication for a psychological condition.

#### Substance Use

The majority of participating legal clinic clients had used alcohol at all, used alcohol to feel the effects, and used cannabis in their lifetime (Table 2). In contrast, the minority of participants had used tobacco, alcohol, or other substances in the 30 days prior to seeking legal services. About one-third had used alcohol to feel the effects, and one-fifth had used cannabis in the prior 30 days.

#### Health Care Utilization

In the six months prior to seeking legal services, most of the legal clinic clients had received treatment in an emergency department (n = 37, 60.7%), most often for medical (n = 28) rather than mental health (n = 9) or substance use (n = 0) concerns (see Table 2). Most had received treatment in outpatient settings (n = 53, 82.0%), most often for medical (n = 27) or mental health (n = 23) rather than substance use (n = 3) concerns. One-quarter of clients (n = 15) had received inpatient treatment in a hospital for medical (n = 9), mental health (n = 5) or substance use (n = 1) concerns, or residential treatment, most often for substance use (n = 10) rather than mental health (n = 4) or medical (n = 1) concerns.

#### Perceived Links Between Legal and Health Concerns

Participants were asked: How much do your legal problems cause your health problems (medical, mental health, and/ or substance use)? Response options were: 0 = not at all, 1 = slightly, 2 = moderately, 3 = considerably, and 4 = extremely. The mean was 1.67 (SD = 1.54). Participants were also asked how much their health problems cause their legal problems, on the same response scale. The mean was 1.66 (1.64). Thus legal clinic clients perceived only a slight to moderate causal link between their legal and health problems.

### Changes in Legal Needs, Housing, Mental Health, and Substance Use Over Time

At follow-up, the mean number of kinds of legal needs reported by participants was 4.4 (SD = 3.8; range = 0–15).

We conducted McNemar tests on changes between baseline and the two-month follow-up on: (1) whether each of the 21 types of legal help (see Figure 1) was needed; (2) whether clients reported having no stable housing arrangement; (3) whether each of seven psychological symptoms (see Table 2) was experienced in the past 30 days; and (4) whether each of 10 substances (see Table 2) was used in the past 30 days. These tests yielded three significant results. Compared to baseline, at follow-up, participants were less likely to report a need for help with VA benefits (85.7% at baseline, 65.3% at follow-up, p = .006), with obtaining ID or other legal documents (34.8%, 15.2%, p = .049), and with housing problems (33.3%, 12.5%, p = .006).

### Amount of and Satisfaction with Legal Services

As shown on Table 3, at follow-up, most participants (69.4%) reported that they did not receive additional help with their legal matters after their initial meeting at the VA-housed legal clinic. Also at follow-up, even more participants (98.0%) reported that they did not receive additional help with criminal legal matters. In keeping with these results, at follow-up, on average, participants had fewer than one additional in-person or phone meeting with legal clinic staff following their first appointment.

On average, at follow-up, clients reported that few of their legal needs were met by legal clinic staff (Table 3). However, clients also reported that, on average, they somewhat agreed with legal clinic staff on goals for their legal needs; that the services they received somewhat helped them deal better with their legal problems; that their legal services somewhat fit with their ideas about what is most helpful to people with legal problems; and that they were mostly satisfied with the legal services they received.

## Discussion

This study obtained data from 61 clients of two VA-housed legal clinics at the time clients were first seeking the clinic’s services. It found that clients represented a vulnerable population in terms of their criminal history and health status. Clients needed a mean of six different types of legal help; 100% needed at least one type of help that brought them to the clinic; 87% needed at least two types of help, and 43% needed at least six types of help. To provide context to these results, a survey of low-income Americans using a nationally-representative sample (Legal Services Corporation, 2017) found that 71% of households had experienced at least one civil legal problem in the past year, 54% had experienced at least two, and 24% had experienced six or more past-year civil legal problems. The civil legal problems faced by these households were most often related to basic needs such as health care access and staying in their homes under safe living conditions. The same survey’s results for households with veterans or military personnel were similar: 71% had at least one civil legal problem in the past year and 21% had six or more such problems in the past year. For this subgroup, common problem areas were related to health, consumer, and employment concerns.

The present study’s data obtained from 49 of the same clients two months later showed that additional use of legal clinics after the first appointment was quite limited, there was little change on clients’ legal needs or health status since baseline, and satisfaction was mixed among clients regarding the legal services they received. Findings suggest the possibility that veteran clients need more intensive legal intervention of longer duration to resolve their legal needs and achieve better housing and health status. However, it will be necessary for future studies to obtain qualitative data by interviewing clients to help determine the extent to which this possibility holds true.

By far the most commonly needed legal service at baseline was help applying for and obtaining VA benefits, which was needed by 87% of participants. Generally, in order to receive VA benefits, the veteran’s character of discharge or service must be under conditions that are not dishonorable, that is, conditions that are honorable or general. However, even individuals receiving undesirable, bad conduct, and other types of dishonorable discharges (n = 9 in our study) may qualify for VA benefits depending on a determination made by VA; accordingly, individuals with these types of discharges may seek legal services to obtain VA benefits. Even veterans who received an honorable or general discharge (n = 52 in our study) may need help obtaining or upgrading VA benefits, due to the complexity of VA’s programs and processes. For example, there are four major VA benefits programs, each with its own procedures: disability compensation, pension programs, free or low-cost VA medical care, and education programs. In keeping with the high need for help with applying for and obtaining VA benefits at baseline, we found that this need decreased between baseline and follow-up. However, even at follow-up, 65% of veterans followed reported that they needed help with applying for and obtaining VA benefits.

Our findings highlight that veterans seeking services at VA-housed legal clinics mainly for civil legal needs are a vulnerable population. In this aspect our findings agree with those of studies on MLPs that embed civil legal services and interprofessional health care teams into health care settings (Paul, Curran, & Tobin, 2017). VA-housed legal clinic clients in the present study had an extensive criminal history in that the majority had been arrested, charged, and incarcerated. They also had multiple health care needs in that the majority had a chronic medical condition for which they were taking prescribed medication, had recently received treatment in an emergency department, and had received psychological treatment due to significant psychological symptoms for which they had taken prescribed medication. In contrast to this sample, a minority of the general population in the US has a chronic medical condition or mental health disorder (Raghupathi & Raghupathi, 2018; Walker & Druss, 2017). This is in keeping with findings that the nonveteran population is less likely than the veteran population to have chronic medical conditions, serious psychological distress, and work limitations (Kramarow & Pastor, 2012). Relative to mental health symptoms, substance use did not appear to be a particular concern among the legal clinic clients in the present sample. Although the majority of clients had used alcohol and cannabis in their lifetime, the minority had used tobacco, alcohol, or cannabis in the month prior to seeking legal services. This is consistent with reports that substance use declines in adulthood after a peak at about age 21; for example, only about 10% of the US population used cannabis by the age of 57 years old (the mean age of participants in this study) (Schulte & Hser, 2014). Our finding of low past-month substance use is also consistent with knowledge that alcohol and drug use should be avoided when it may exacerbate chronic medical conditions and complicate effective management of these conditions (Timko et al., 2016), which were common in our sample.

Despite their high rates of medical and psychological problems, VA-housed legal clinic clients perceived only a slight to moderate causal link between their legal and health concerns. This stands in contrast to legal and policy experts’ evidence that law is a critical determinant of health (Teitelbaum, Theiss, & Boufides, 2019), and to veterans in Veterans Treatment Courts who report a clear connection between their criminal legal needs and their psychological problems (Herzog, Ferdik, Scott, Denney, & Conklin, 2019). Study participants’ views on the lack of a legal-health connection may be related to our findings that for the most part, improvement was not demonstrated from baseline to follow-up on legal needs, housing status, psychological symptoms, or substance use. In addition, participants still reported a mean of four remaining legal needs at follow-up.

Two months is likely too short a time to achieve health benefits from addressing civil legal problems, especially given that clients had little contact with clinic staff after the initial appointment. In this regard, although many legal issues may be resolved with brief services provided by staff who are not attorneys, civil suits, for example, can take several years to be resolved. Renner and Harley’s (2018) one-year study did not find an association between type or amount of civil legal services received and participants’ (women who had experienced domestic violence) improvement on psychological well-being. Moreover, individuals with civil legal needs often reported that their health suffered while they were engaged in attempting to resolve those needs (Pleasence, Balmer, Buck, O’Grady, & Genn, 2004). More research, both quantitative and qualitative, is needed to examine the duration of civil legal problems and of their processes of resolution, and when positive effects may occur after problem resolution in terms of gaining access to services and gaining better health. Possibly, a longer duration and intensity of legal services would favorably impact clients’ satisfaction, which in this study was positive but moderate. Generally, veterans’ stronger identification as a veteran (the individual has social bonds with other veterans and positive feelings about being grouped with veterans) is associated with having stronger beliefs that legal agencies are fair, based on shared values, and should be obeyed (Gallagher & Ashford, 2019).

### Limitations

Limitations of this study included its small sample gathered from only two VA-housed legal clinics. In addition, we conducted multiple McNemar tests without correction and relied on clients’ self-reports. This study used a longitudinal design to examine client characteristics, improvement on legal needs, and satisfaction with legal services. Although it did not aim to predict outcomes of obtaining legal clinic help using multivariate models, future studies with larger samples from a broader range of clinics should consider using this method to investigate, for example, multiple predictors of clients’ additional contact with their clinic, or of greater satisfaction with the help received.

## Conclusions

This study found that clients of VA-housed legal clinics are likely to represent a vulnerable population and to arrive seeking help with several legal needs. In the two months that followed initial service provision to clients, there was little additional clinic contact, and a modest reduction in legal needs but none in clients’ housing, psychological symptoms, or substance use. Other evidence suggests that limited client-clinic contact is likely attributable to under-resourcing of VA-housed legal clinics. Specifically, our survey of VA-housed legal clinics found that about one-third of clinics did not have dedicated and adequate space, that most clinic staff members were unpaid, and that most clinics lack funds to fully serve all veterans seeking services (Timko et al., 2019). Such findings suggest that VA and community agencies should enact policies that expand and fund veterans’ legal services and health system interactions to better address health vulnerabilities and improve outcomes (Tsai et al., 2017b). Goals would including having a civil legal aid clinic in every VA medical center and strengthening national network ties among VA-housed legal clinics to help them share best practices for identifying funding streams, attorney and law student recruitment procedures, staff trainings, client intake and outcome monitoring systems, and other aspects of clinic management activities. Sharing of funding resources, procedures, trainings, and systems could be accomplished with an online toolkit and by collaborating with other veteran legal aid organizations (Sandman, 2019).

Another way to expand civil legal services is to implement alternate ways of providing them, such as connecting clients with clinic staff through internet video conferencing (Keith, 2016). Leveraging technology to provide legal services can potentially increase a clinic’s ability to serve more clients for a longer duration, including those in rural or isolated areas or unable to travel to a clinic. Such expansion may help clients to experience VA-housed legal clinics as responsive to their needs and circumstances.

## Figures and Tables

**Table 1:** Baseline demographic characteristics of VA-housed legal clinic clients (n = 61).

	% (n)	M(SD)

Male	90.2 (55)	
Age		56.8 (13.9)
Hispanic or Latino	24.6 (15)	
Race		
White	45.0 (27)	
Black	21.7 (13)	
Asian	3.3 (2)	
Native Hawaiian, Pacific Islander	6.7 (4)	
Other	23.3 (15)	
Years of education		14.2 (2.1)
Employed	32.8 (20)	
Monthly income ($)		1,098 (1,500)
Marital status		
Currently married	18.0 (11)	
Formerly married	55.8 (34)	
Never married	26.2 (16)	
Usual living arrangement		
With family or friends	34.4 (21)	
Alone	32.8 (20)	
Controlled environment	6.6 (4)	
No stable arrangement	26.2 (16)	
Military branch		
Army	49.2 (30)	
Navy	23.0 (14)	
Air Force	13.1 (8)	
Marine Corps	9.8 (6)	
National Guard	0.0 (0)	
>1 branch	4.9 (3)	
Number of years in the military		5.1 (4.1)
Deployed to Iraq or Afghanistan since 9/11/2001	11.5 (7)	
Ever deployed to combat zone Type of military discharge*	34.4 (21)	
Honorable	73.8 (45)	
General	11.5 (7)	
Bad conduct	4.9 (3)	
Other than honorable	9.8 (6)	
Received disciplinary action while in the military	55.7 (34)	

* Honorable discharge: Military service member received a good or excellent rating for service time. General discharge: Military service member received a satisfactory rating. Bad conduct discharge: Enlisted military member received court-martial due to punishment for bad conduct; virtually all veterans benefits are forfeited. Other than honorable discharge: Military service member engaged in an action such as security violation, use of violence, or conviction in a civilian court with a sentence including prison time; cannot re-enlist in the armed forces except under very rare circumstances; usually cannot receive veterans’ benefits.

**Table 2:** Baseline psychosocial characteristics of VA-housed legal clinic clients (n = 61).

***Criminal justice involvement***	**% (n)**	**M (SD)**

Awaiting charges, trial, sentence	4.9 (3)	
On parole or probation	8.2 (5)	
In a treatment court	3.3 (2)	
Ever arrested and charged	82.0 (50)	
Has been incarcerated	60.7 (37)	
Number of months incarcerated (if ever)		25.8 (42.2)
***Health***		

Has chronic medical condition	78.7 (48)	
Takes prescribed medication for medical condition	65.6 (40)	
Ever received psychological treatment	80.3 (49)	
Receives a pension for a psychiatric disability	5.0 (9)	
**Had a significant period of time experiencing**	**Lifetime**	**Past 30 days**
	**% (n)**	**% (n)**

Serious depression	91.8 (56)	67.2 (41)
Serious anxiety	91.8 (56)	72.1 (44)
Hallucinations	37.7 (23)	23.0 (14)
Trouble understanding, concentrating, remembering	73.8 (45)	55.7 (34)
Trouble controlling violent behavior, rage, violence	41.0 (25)	14.8(9)
Serious thoughts of suicide	59.0 (36)	14.8 (9)
Attempted suicide	37.7 (23)	3.3 (2)
Taking prescribed medication for psychological condition	73.8 (45)	57.4 (35)
***Substance use***		

Tobacco	not asked	37.7 (23)
Alcohol (at all)	86.9 (53)	34.4 (21)
Alcohol (to feel effects)	59.0 (36)	13.1 (8)
Heroin	14.8 (9)	0 (0)
Opiold/analgesics (not prescribed)	9.8 (6)	0 (0)
Barbiturates	13.1 (8)	0 (0)
Sedatives/Hypnotics/Tranquilizers	13.1 (8)	8.2 (5)
Cocaine	42.6 (26)	6.6 (4)
Amphetamines	29.5 (18)	6.6 (4)
Cannabis	57.4 (35)	19.6 (12)
Hallucinogens	18.0 (11)	0 (0)
Inhalants	3.3 (2)	0 (0)
***Baseline prescribed medication use***		

Methadone, buprenorphine, naltrexone	14.9 (9)	14.8 (9)
Opioids/analgesics	31.1 (19)	14.8 (9)
***Health care utilization***	**% (n)**	
**In the past 6 months,**		

Stayed overnight in the hospital	24.5 (15)	
Received treatment in the emergency room	60.7 (37)	
Received residential treatment	24.5 (15)	
Received outpatient treatment	82.0 (5)	

**Table 3:** VA-housed legal clinic clients’ experiences at follow-up (n = 49).

After your visit with the VA-housed legal clinic, did you receive additional help with civil legal matters?	% (n)	M (SD)

Yes, from the legal clinic staff	14.3 (7)	
Yes, from a referral received at the legal clinic	14.3 (7)	
Yes, other	2.0 (1)	
No	69.4 (34)	
After your visit with the VA-housed legal clinic, did you receive additional help with criminal legal matters? Yes, from the legal clinic staff	0 (0)	
Yes, from a referral received at the legal clinic	2.0 (1)	
Yes, other	0 (0)	
No	98.0 (48)	
Number in-person meetings with VA-housed legal clinic staff		47 (.94)
Number phone meetings with VA-housed legal clinic staff		.44 (1.00)
Extent to which VA-housed clinic staff met your legal needs (0 = none; 1 = few; 2 = most; 3 = almost all)		1.32 (1.20)
Extent to which you and legal clinic staff agreed on goals for your legal needs (0 = totally disagreed; 1 = disagreed somewhat; 2 = agreed somewhat; 3 = totally agreed)		2.39 (.99)
Services you received help you deal better with your legal problems (0 = no, made things worse; 1 = no, didn’t help; 2 = yes, somewhat; 3 = yes, a great deal)		2.07 (.915)
How legal services fit with your ideas about what is most helpful to people with legal problems (0 = didn’t fit at all; 1 = fit a little; 2 = fit somewhat; 3 = fit very well)		2.02 (1.03)
How satisfied with legal services received (0 = very dissatisfied; 1 = indifferent or mildly dissatisfied; 2 = mostly satisfied; 3 = very satisfied)		1.91 (1.05)
